# Supplementary figures and images for: Spatiotemporal expansion of human brucellosis in Shaanxi Province, Northwestern China and model for risk prediction
Source: PeerJ. 2020 Oct 19;8:e10113. doi: 10.7717/peerj.10113 (PMC7580622; doi:10.7717/peerj.10113)

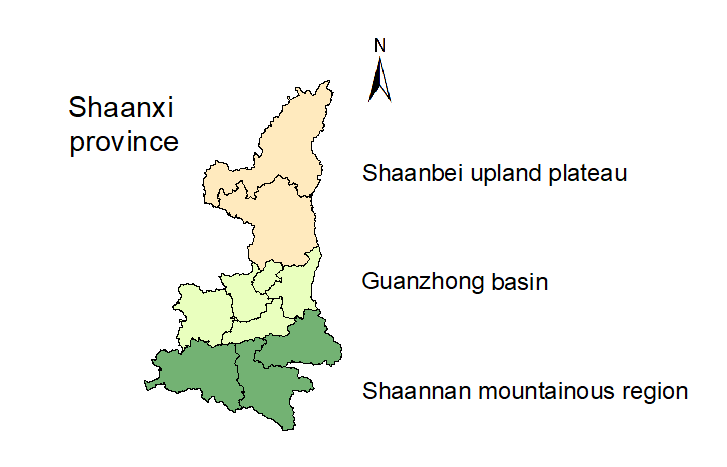

Supplement: Figure S1 — The map was created by Zurong Yang in ArcGIS 10.1 Software, ESRI Inc., Redlands, CA, USA, (https://www.arcgis.com/index.html). [file peerj-08-10113-s001.png]

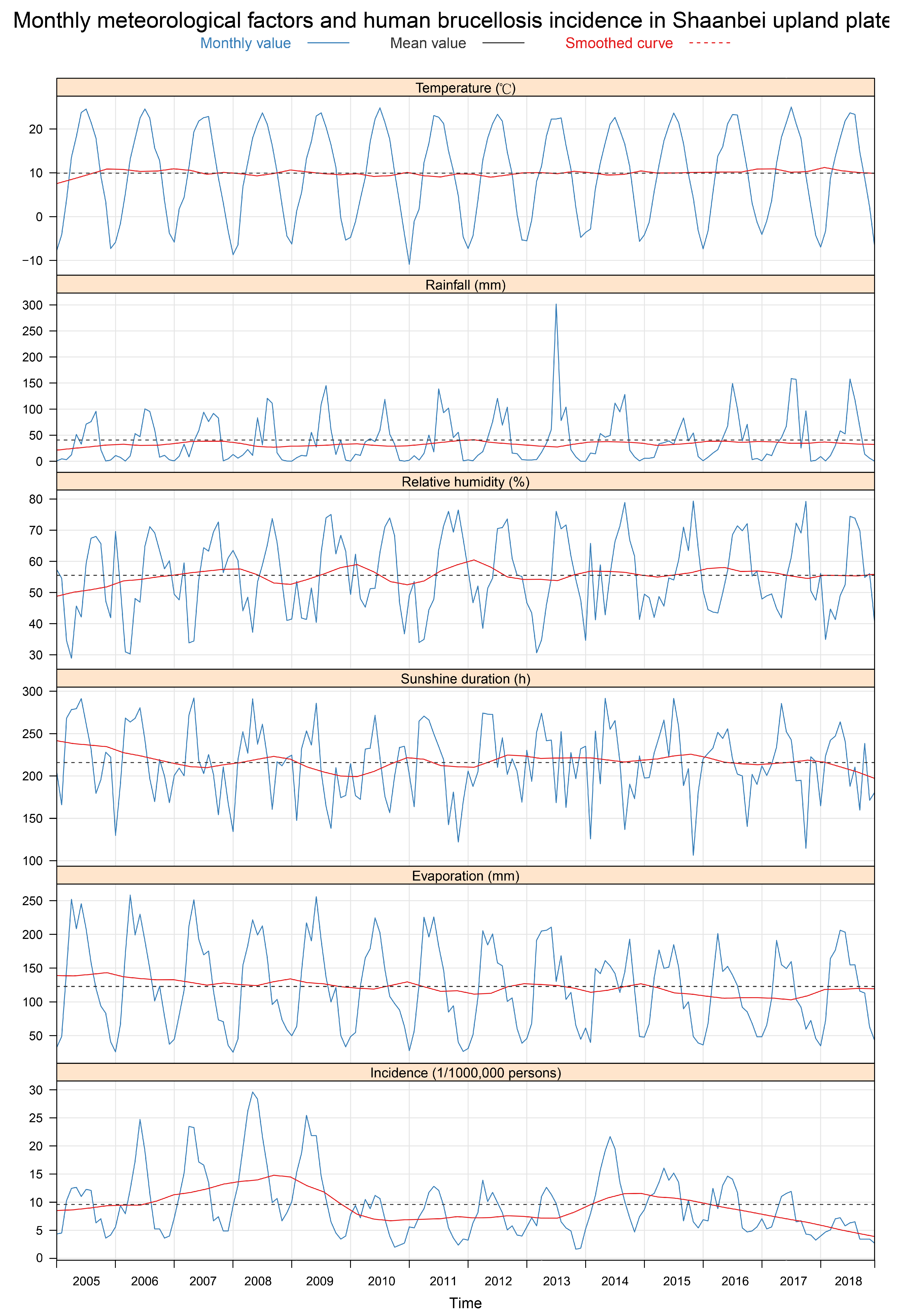

Supplement: Figure S2A [file peerj-08-10113-s002.png]

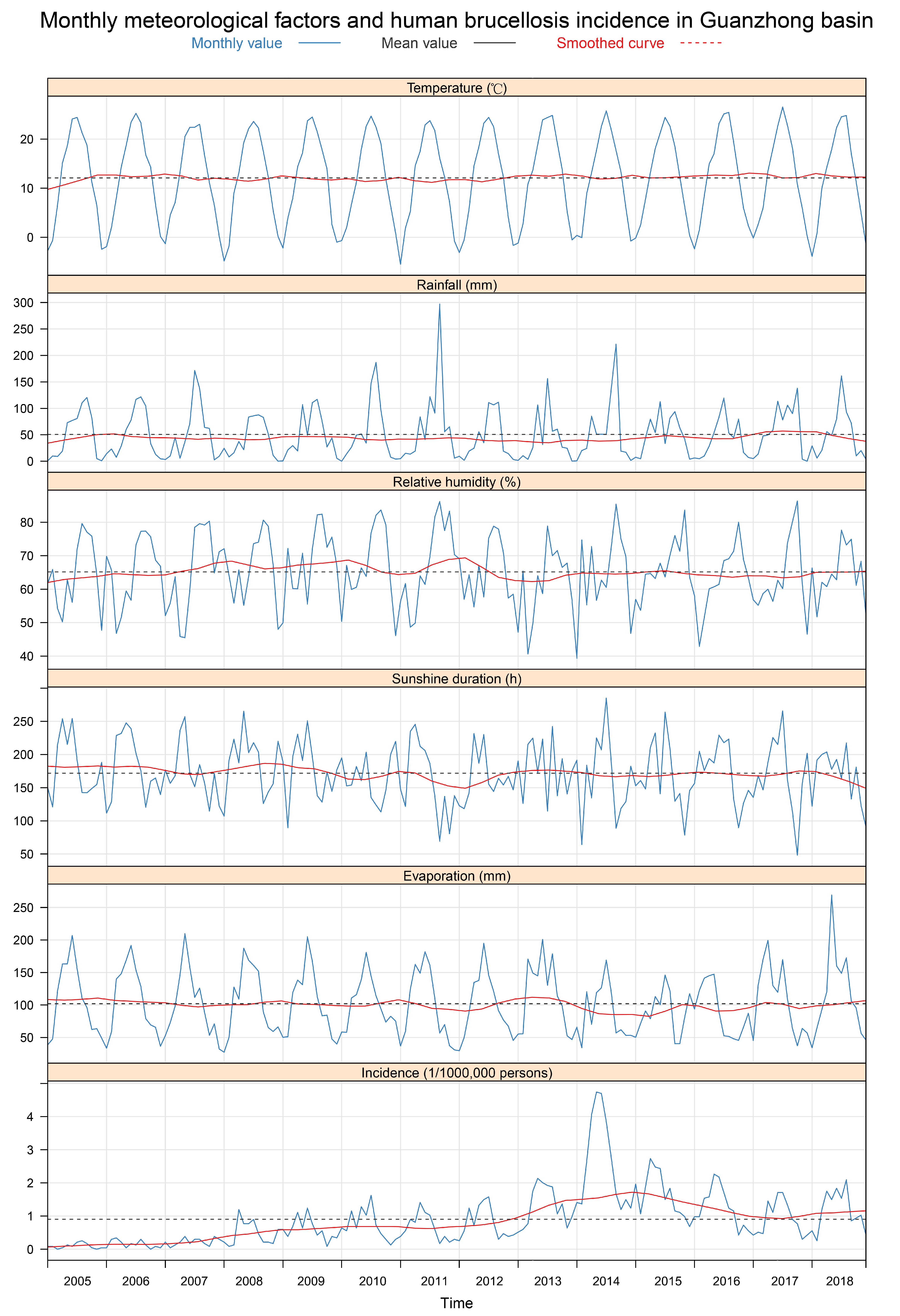

Supplement: Figure S2B [file peerj-08-10113-s003.png]

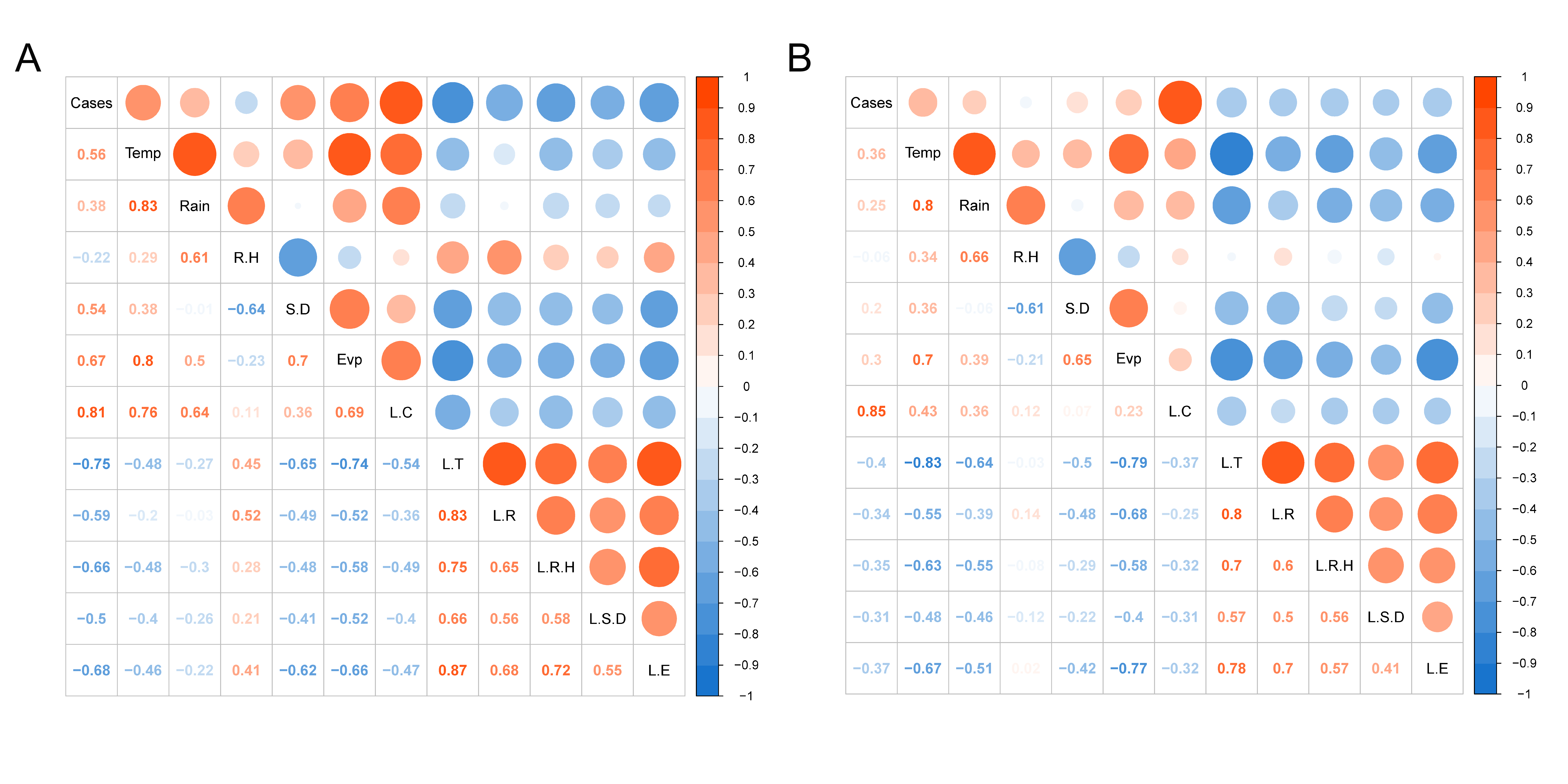

Supplement: Figure S3 — Cases: monthly number of human brucellosis; Temp: monthly mean temperature; Rain: monthly mean rainfall; R.H: monthly mean relative humidity; S.D: monthly cumulative sunshine duration; Evp: monthly cumulative evaporation; L.C: lag of monthly number of human brucellosis; L.T: lag of monthly mean temperature; L.R: lag of monthly mean rainfall; L.R.H: lag of monthly mean relative humidity; L.S.D: lag of monthly cumulative sunshine duration; L.E: lag of monthly cumulative evaporation. [file peerj-08-10113-s004.png]
